# Complex I deficiency remains the most frequent cause of Leigh syndrome spectrum

**DOI:** 10.1093/braincomms/fcae470

**Published:** 2024-12-23

**Authors:** Shamima Rahman

**Affiliations:** Mitochondrial Research Group, Genetics and Genomic Medicine Department, UCL Great Ormond Street Institute of Child Health, 30 Guilford Street, London WC1N 1EH, UK

## Abstract

This scientific commentary refers to ‘Biallelic *NDUFA13* variants lead to a neurodevelopmental phenotype with gradual neurological impairment’, by Kaiyrzhanov *et al*. (https://doi.org/10.1093/braincomms/fcae453).

**This scientific commentary refers to ‘Biallelic *NDUFA13* variants lead to a neurodevelopmental phenotype with gradual neurological impairment’, by Kaiyrzhanov *et al*. (https://doi.org/10.1093/braincomms/fcae453).**

In this issue of *Brain Communications*, Kaiyrzhanov *et al.*^1^ report nine new families with pathogenic variants in *NDUFA13*, encoding an accessory subunit of mitochondrial respiratory chain complex I. All but one of the 10 affected individuals presented with clinical features in the Leigh syndrome spectrum. This adds to the evidence that complex I deficiency is the most common form of Leigh syndrome, an observation first made nearly 30 years ago, when it was noted that approaching 20% of a large cohort of patients with Leigh syndrome had complex I deficiency.^2^

Previously, biallelic *NDUFA13* variants were initially reported in two sisters with early onset hypotonia, dyskinesia and optic neuropathy^3^ and subsequently in a single case with Leigh syndrome associated with mild hypertrophic cardiomyopathy and progressive spastic tetraparesis.^4^ One patient in the *NDUFA13* cohort described by Kaiyrzhanov *et al.* presented with skeletal dysplasia and glaucoma. The very different clinical manifestations in this case compared to all other patients reported with pathogenic *NDUFA13* variants suggests that *NDUFA13* may not be disease-causing in this individual, or that the reported variant is hypomorphic.

Denis Leigh reported the characteristic neuropathology that came to be eponymously associated with him in 1951. The brain of a 7-month-old infant who died from respiratory failure after a 6-week illness characterized by somnolence with prominent visual impairment was found to have necrotic lesions with intense capillary proliferation, gliosis, severe neuronal loss and relative sparing of astrocytes.^5^ Leigh syndrome is now recognized to be the most frequent presentation of primary mitochondrial disease in childhood. This phenotype has been linked to at least 113 genes, with heterogeneous aetiologies, including deficiencies of subunits and assembly factors of all five oxidative phosphorylation enzyme complexes, defects of mitochondrial DNA maintenance and expression, and disorders of mitochondrial membranes, dynamics, quality control, toxicity, cofactor biosynthesis and transport and metabolism of vitamins.^6^ The ClinGen consortium aims to evaluate gene–disease relationships (GDRs) for a large range of monogenic disorders. Gene curations are performed by a team of biocurators using a regimented standard operating procedure approved by both the ClinGen leadership and the disease area expert panel. Of the 113 Leigh syndrome gene curations, 22 were for complex I structural subunits and eight for complex I assembly factors.^6^ Nine of the complex I subunits had a definitive GDR for Leigh syndrome, while there was a moderate GDR for nine subunits, and limited evidence for the final four subunits (Fig. 1). *NDUFA13* was previously curated to have a moderate GDR for Leigh syndrome, but the nine new cases reported by Kaiyrzhanov *et al.* will likely elevate this to a definitive GDR.

**Figure 1:**
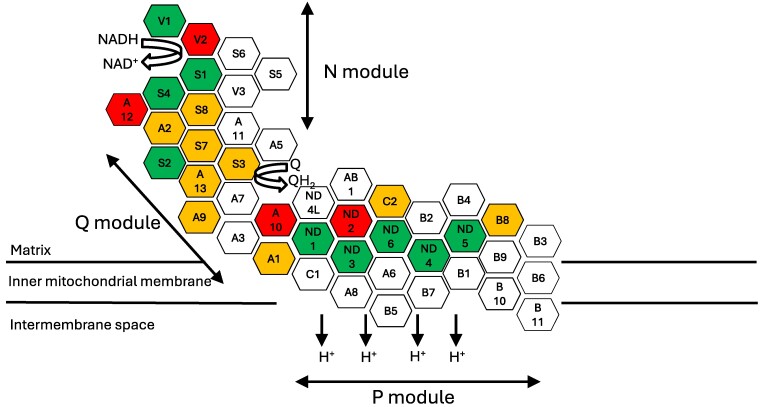
**Complex I structural subunits associated with Leigh syndrome.** Cartoon to illustrate the structure of complex I, and association of structural subunits with Leigh syndrome spectrum. Green denotes definitive gene–disease relationship (GDR), amber a moderate GDR and red a limited GDR.

Consideration of the clinical features of all patients now reported with *NDUFA13* variants revealed early faltering growth in 9/13 cases, a frequent feature in Leigh syndrome of many causes. Of the patients now reported with NDUFA13 deficiency, 84% had oculomotor abnormalities and 54% had optic atrophy, possibly representing a more specific finding, although eye involvement is frequently observed in other causes of complex I deficiency.^7^ The reasons for prominent ophthalmological involvement in complex I deficiency remain unknown, as do the precise pathomechanisms underlying the focal necrotic lesions defining Leigh syndrome. Energy deficiency and oxidative stress have been postulated but not proven. More recently, the intriguing possibility of direct oxygen toxicity has been mooted.^8^

Kaiyrzhanov *et al.* suggest that there may be a characteristic neurological profile of NDUFA13 deficiency, with spasticity (ten cases) and ataxia (eight patients). Spasticity and ataxia are relatively frequent findings in Leigh syndrome (each associated with 49 genes in the Leigh map diagnostic resource, https://www.vmh.life/#leighmap), but the choreoathetosis and dyskinesias reported in seven NDUFA13 deficient patients may be more discriminating (associated with 11 and two genes, respectively, in Leigh map).^9^ Seizures, reported in six cases, also occur relatively often in complex I deficiency and therefore are not a selective feature. Little detail is provided about the nature of the seizures or any EEG abnormalities in this cohort, so it is not possible to comment on whether specific seizure types are associated with *NDUFA13* disease.

From the 1990s, magnetic resonance imaging of the brain, specifically T2 hyperintensities variably involving the basal ganglia, midbrain, brainstem, cerebellum and spinal cord, in a compatible clinical context with evidence of mitochondrial dysfunction, came to supersede post-mortem brain examination in the diagnosis of Leigh syndrome.^2^ Brain imaging of the NDUFA13 deficient cohort revealed prominent involvement of the substantia nigra (11/12 cases) and bilateral optic nerve atrophy (8/12). Imaging of one Leigh syndrome cohort demonstrated substantia nigra lesions in 60%,^10^ while a neuropathological review revealed substantia nigra lesions in 95% of cases,^11^ suggesting that substantia nigra involvement is not specific for *NDUFA13* disease. A study analysing brain transcriptomic data available in the United Kingdom Brain Expression Consortium (UKBEC) network showed that 64% of genes linked to Leigh syndrome are enriched in the substantia nigra, which may explain the predilection for the substantia nigra in NDUFA13 deficiency and other causes of Leigh syndrome.^12^ Basal ganglia lesions, a frequent finding in Leigh syndrome, were only observed in three individuals with NDUFA13 deficiency. Of note, two individuals were reported to have normal MRI brain images in the first year of life (at 6 and 9 months, respectively), suggesting that a normal brain MRI in infancy may be falsely reassuring in individuals with NDUFA13 deficiency (and possibly other causes of complex I deficiency); imaging should be repeated if there is ongoing clinical suspicion.

NDUFA13, also known as ‘genes associated with retinoid-interferon mortality-19’ (GRIM-19), was first identified as a cell death regulatory protein activated by interferon beta and retinoic acid. Subsequently, numerous roles unrelated to its function in complex I have been attributed to this protein, including inhibition of STAT3 activity, tumour suppression and autophagy.^13^ Recent studies have suggested a neuroinflammatory component of primary mitochondrial disease, with abnormal interferon stimulated gene expression emerging as a potential biomarker of primary mitochondrial disease.^14^ Given its dual roles in complex I structure and interferon signalling, one could conjecture that NDUFA13 may turn out be a key component of the signalling pathway between primary mitochondrial disease and interferon dysregulation.

## Data Availability

No new data were generated or analysed in support of this scientfic commentary.
